# Editorial: 15 Years of Frontiers in Cellular Neuroscience: astrocytes in brain disease

**DOI:** 10.3389/fncel.2024.1374172

**Published:** 2024-02-02

**Authors:** Seok-Geun Lee

**Affiliations:** Department of Biomedical Science and Technology, BioNanocomposite Research Center, Kyung Hee University, Seoul, Republic of Korea

**Keywords:** brain, central nervous system, astrocytes, brain tumor, neurodegenerative diseases

Astrocytes, once considered mere support cells for maintaining the brain's microenvironment, have now taken center stage in understanding the complexities of brain health and disease (Lee et al., 2022; Verkhratsky et al., 2023). Recent research has uncovered their intricate roles, particularly in regulating glutamate, a major excitatory neurotransmitter in the central nervous system (CNS) (Kim et al., 2011; Lee et al., 2022; Verkhratsky et al., 2023). In neurodegenerative diseases, astrocytes exhibit neurotoxic phenotypes that can exacerbate disease progression, playing crucial roles in neurogenesis, synaptogenesis, and maintaining extracellular space homeostasis (Kim et al., 2011; Lee et al., 2022; Verkhratsky et al., 2023). Pathogenic alterations in astrocytes, involving neurotransmitters, cell communication, receptors, and signaling pathways, and changes in astrocyte size and number, are central to various neurological disorders (Kim et al., 2011; Lee et al., 2022; Verkhratsky et al., 2023). Astrocytes are also implicated in brain tumor progression, particularly in astrocytomas, the most common primary brain tumor within gliomas (Robert and Sontheimer, 2014; Krawczyk et al., 2022; Verkhratsky et al., 2023). Brain tumors induce neurodegeneration through mechanisms like reduced EAAT2 or elevated xCT expression, leading to increased extracellular glutamate levels, resulting in glutamate excitotoxicity, neuronal damage, brain swelling, and tumor-associated seizures (Lee et al., 2011, 2013; Robert and Sontheimer, 2014; Seo and Park, 2020; Krawczyk et al., 2022; Verkhratsky et al., 2023).

This editorial explores the insights from three reviews and one research article, each shedding light on different facets of astrocyte biology and their profound impact on various neurological disorders.

The journey into astrocyte biology begins with a mini-review on the role of non-neoplastic astrocytes in brain tumor progression (Catalano et al.). Contrary to their traditional portrayal as mere structural support, astrocytes are now recognized as key players in the development and advancement of primary and secondary brain tumors. The interaction between astrocytes and different tumor types, such as glioblastoma and metastatic tumors, reveals a complex web of mechanisms influencing tumor growth, invasion, angiogenesis, and immune response. Astrocyte-tumor crosstalk takes center stage, involving secreted molecules, extracellular vesicles, gap junctions, and tunneling nanotubes. The modulation of astrocyte-derived products as potential therapeutic targets to impede tumor progression and recurrence opens new avenues for intervention. The intricate dance of chemokines and their receptors in glioma-astrocyte interactions and the impact of glioma-derived exosomes on astrocyte function provide further layers of understanding. Gap junctions and tunneling nanotubes forming direct intercellular connections between glioma cells and astrocytes showcase the physical dimension of this interaction, influencing the tumor microenvironment and immune responses. The authors also discuss the potential use of astrocytes or their derived products to counteract brain tumor progression and recurrence.

Transitioning to the broader landscape of reactive astrogliosis, the second article (Matusova et al.) provides a panoramic view of the heterogeneous responses of astrocytes to CNS disturbances. Single-cell transcriptomics unravels the intricate tapestry of reactive astrocytes across various neuropathologies, from neuroinflammation to neurodegeneration. The review dissects gene expression patterns, morphological variations, and functional dynamics of reactive astrocytes, highlighting their roles in disease progression. In neurodegenerative diseases like Alzheimer's, Parkinson's, amyotrophic lateral sclerosis, and Huntington's, commonalities and distinctions in reactive astrocyte responses are explored. Temporal and spatial dynamics of astrocyte reactivity in acute injuries offer insights into beneficial and detrimental effects on tissue repair and regeneration. Other pathological conditions like multiple sclerosis, experimental autoimmune encephalomyelitis, and aging reveal molecular signatures and functional implications of reactive astrocyte subtypes. The interplay between astrocyte-microglia interactions and astrocyte heterogeneity emerges as a key modulator of neuroinflammation and neurodegeneration.

Zooming into the realm of CNS demyelinating diseases, the third article (Tan et al.) navigates the multifaceted roles of astrocytes in maintaining myelin integrity and function. Astrocytes contribute to CNS homeostasis and can influence demyelination, either positively or negatively, depending on their subtype, and environment. The dichotomy of astrocyte behavior, either promoting or inhibiting remyelination, unveils a complex regulatory network. They can produce and release various factors that modulate immune cell activation, myelin debris clearance, and recruitment and differentiation of oligodendrocyte precursor cells (OPCs). The interactions between astrocytes, oligodendrocytes, OPCs, and synapses are explored as potential therapeutic targets for myelin regeneration and repair of synaptic dysfunction in demyelinating diseases. The review (Tan et al.) extends its reach into various demyelinating diseases, such as traumatic injury, stroke, Parkinson's, and Alzheimer's disease. This review (Tan et al.) concludes by emphasizing the need for comprehensive research to comprehend the complex roles of astrocytes in health and disease, discussing the challenges and opportunities in developing astrocyte-focused interventions and highlighting the potential of astrocytes not only as a key player in the progression of demyelinating diseases but also as promising targets for therapeutic strategies.

In the final exploration, the spotlight shifts to the intricate world of astrocytic coupling and uncoupling under brief metabolic stress (Eitelmann et al.). The study investigates how astrocytes respond to transient metabolic stress, mimicking conditions in the ischemic penumbra, through Ca^2+^-dependent mechanisms. In a carefully designed *in situ* model, the findings reveal that brief metabolic stress induces a rapid and reversible uncoupling of astrocytes, mediated by a Ca^2+^/calmodulin-dependent mechanism. This uncoupling, suggested to limit and reduce cellular damage in the ischemic penumbra, introduces a protective aspect to astrocyte function. The results hint at the dynamic adaptability of astrocytes to transient metabolic challenges, highlighting the intricate regulatory mechanisms governing their coupling dynamics.

As we weave through the insights of these four articles, a symphony of astrocyte biology emerges. No longer confined to the sidelines, astrocytes take center stage in orchestrating the delicate balance of brain health and navigating the tumultuous terrain of neurological disorders. From their intricate involvement in tumor progression, through the nuanced responses of reactive astrogliosis revealed by single-cell transcriptomics, to their pivotal role in maintaining myelin integrity in demyelinating diseases, and finally, their dynamic adaptability under brief metabolic stress—astrocytes emerge as conductors of the neurological orchestra. Thus, a comprehensive understanding of astrocyte biology, from molecular intricacies to dynamic interactions in health and disease, is essential for unlocking the therapeutic potential within these enigmatic cells.

In conclusion, the exploration of astrocyte biology signifies a paradigm shift in our understanding of the brain's intricacies. As we unravel the mysteries of astrocytes, we pave the way for a new era in neuroscience, where these once-overlooked cells emerge as protagonists in the narrative of brain health and disease.

## Author contributions

S-GL: Conceptualization, Investigation, Writing—original draft, Writing—review & editing.
